# Advances in Honey Bee Virus Research

**DOI:** 10.3390/v12101149

**Published:** 2020-10-10

**Authors:** Nor Chejanovsky

**Affiliations:** Entomology Department, Inst. of Plant Protection, The Volcani Center, Rishon Lezion 7528809, Israel; ninar@volcani.agri.gov.il

The sudden collapse of honey bee colonies in California in 2005 and alarming reports about significant colony losses in the U.S. and Europe have attracted the renewed attention of researchers and the general public to the role played by honey bee viruses in this process. Thus, research focused on the elucidation of the genomic sequences of previously identified and new honey bee-associated viruses, their pathogenicity at the individual and colony levels, their structure, their interactions with other pathogens, and their relationship with the host, many times emerging from covert-asymptomatic to overt-symptomatic infections following stress challenges, has recently been conducted. These efforts have resulted in new and substantial knowledge.

The aim of this Special Issue of *Viruses* is to highlight recent significant advances in this area of virology to provide an updated integrated picture of honey bee-associated viruses in order to improve, facilitate, and encourage further innovative research to broaden our understanding in this fascinating field. 

The first part of this issue is dedicated to three studies reporting the development of honey bee virus clones and vectors that enable follow-up and reverse genetic-based studies. Ryabow et al. [1] and Gusachenko et al. [2] describe the development of Deformed wing virus (DWV) vectors that allow the expression of the green fluorescent protein (GFP) during infection of larvae and pupae [1,2], enabling one to trace the virus infection in the infected individual and its localization in specific tissues. Tagged viruses that were developed using a reverse genetics system enabled the detection of DWV replication in *Varroa destructor* mites [2]. Jin et al. [3] took a similar approach to develop a genetically stable GFP clone of another important iflavirus, the sacbrood virus (SBV). The above studies pave the way to gain fundamental knowledge on the pathogenesis and transmission of DWV and SBV at the molecular level.

In this respect, it is worth noticing that cell culture systems capable of sustaining virus replication proved to be powerful tools in animal and insect virology to study virus replication, and its molecular components and their role in infection and spreading it. However, cell lines capable of sustained replication of honey bee viruses are very scarce. Guo et al. [4] provide a comprehensive review on the status of *A. mellifera* cell lines, as well as other cell lines that might be helpful, including a description of ways to develop new cell lines. Moreover, Tal and Chejanovsky [5] show that a heterologous Lepidopteran cell line is able to sustain DWV replication and to yield a virus infectious to *A. mellifera* pupae.

The interaction between the invading virus and the host's defense mechanism determines the outcome of the infection. McMenamin et al. [6] provide evidence that the heat-shock response is an antiviral response in *A. mellifera* by studying the response of heat-shocked honey bees towards a model virus, Sindbis-GFP. They show that six heat-shock protein-encoding genes and three immune genes were differentially expressed in heat-shocked and/ or virus-infected bees [6].

The effect of pesticides as stressors affecting virus infections in *A. mellifera* is reviewed by Harwood and Dolezal [7]. They examine how pesticides and viruses may interact in additive or synergistic ways to affect honey bee health and the complexity of these interactions.

Transmission of viruses in the colony can highly influence its health. The impact of horizontal transmission of the Black queen virus (BQCV) to queens and adults in *A. mellifera* is addressed in a study by Al Naggar and Paxton [8], and an illustration of the interaction between viruses and queens in sublethal infections is presented by Amiri et al. [9]. 

Many studies were published on the extensive damage to *A. mellifera* colonies that results from the complex interaction between honey bees, *Varroa destructor* mites, and viruses. What could we expect to be the outcome of the above interaction in the absence of DWV? Roberts et al. [10] provide us with a clue to the tolerance of honey bees to the Varroa mite in Papua New Guinea by studying their viral landscape using new generation sequencing and showing that DWV was absent in these bees. 

Finally, transmission of honey bee viruses to other insects, and vice versa, can have important ecological effects. Two papers that deal with viruses from Vespidae (*Vespula pensylvanica* and the invasive species *Vespula vellutina*) close this issue, broadening our knowledge in this aspect [11,12].

I do hope that this Special Issue of Advances in Honey Bee Virus Research provides substantial new information and encourages novel research to understand honey bee viruses’ infections at the molecular level in the individual and in the colony. 
